# One-pot domino syntheses of 3-alkyl-3-*N*-substituted aminobenzofuran-2(3*H*)-ones based on alkali-promoted Michael addition and lactonization

**DOI:** 10.1098/rsos.231510

**Published:** 2024-02-14

**Authors:** Xiao-Hui Chu, Na Gao, Wei Wang, Zhong Zheng, Jin-Jun Wang

**Affiliations:** ^1^ College of Food & Biological Engineering, Yantai institute of Technology, 100 Gangcheng East Street, Laishan District, Yantai 264005, People's Republic of China; ^2^ Yantai Key Laboratory of Special Medical Food, Industrial Research Institute of Special Food, Yantai 264005, People's Republic of China; ^3^ College of Chemistry and Chemical Engineering, Yantai University, Yantai 264005, People's Republic of China

**Keywords:** benzofuran-2(3*H*)-one, aryl glycine ester, one-pot domino syntheses, Michael addition, lactonization, stereoselectivity

## Abstract

In this paper, a novel cascade reaction of caesium carbonate-promoted Michael addition and lactonization for the one-pot synthesis of 3-alkyl-3-*N*-substituted aminobenzofuran-2(3*H*)-one derivatives has been established based on the screening of the alkaline reagents and optimization of reaction conditions, in which the *N*-substituted (*ortho*-hydroxy)aryl glycine esters were used as the Michael donors to react with different *α*, β-unsaturated carbonyl compounds. In the case of using the asymmetric starting material, the epimers could be successfully separated by conventional chromatography. In addition, plausible mechanisms were suggested and the absolute configuration of the epimer was analysed. All the chemical structures of unreported benzofuran-2(3*H*)-one derivatives were characterized by ^1^H nuclear magnetic resonance (NMR), ^13^C NMR, IR and high-resolution mass spectrometry (HRMS).

## 1. Introduction

**Figure 1.**
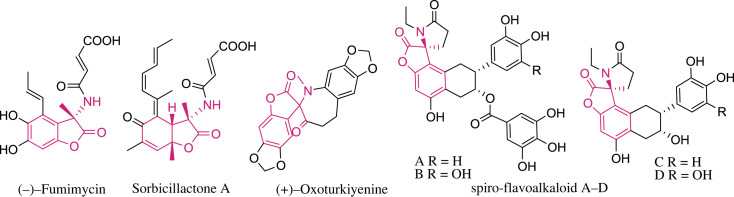
Representative biologically active 3-alkyl-3-*N*-substituted aminobenzofuran-2(3*H*)-ones.

3,3-disubstituted benzofuran-2(3*H*)-ones, also known as 3,3-disubstituted 2-coumaranones, are commonly found in various medically effective natural products [1–3]. They serve as important scaffolds for biologically active compounds with interesting cytotoxic and pharmacological properties [4,5]. Furthermore, they play a role in functionalized organic molecules used in materials, supramolecular and polymer chemistry [6–9]. Many of these compounds feature an aminated quaternary stereocentre at the C3 position of the 2-coumaranone ring. Examples include (-)-fumimycin [10] (antibacterial), sorbicillactone A [11] (antileukemic), (+)-Oxoturkiyenine A [12,13] (transmembrane protease serines and cathepsin L inhibitor), and recently identified spiro-flavoalkaloids [14] (α-Glucosidase inhibitor) (figure 1). Given the synthetic and biological relevance of these 2-coumaranones, significant attention has been devoted to constructing suitable structural motifs and investigating their potential biological activities. Therefore, the development of efficient methods for the synthesis of 3-alkyl-3-*N*-substituted aminobenzofuran-2(3*H*)-ones is an attractive area of research in organic synthesis [15–17].

**Scheme 1.**
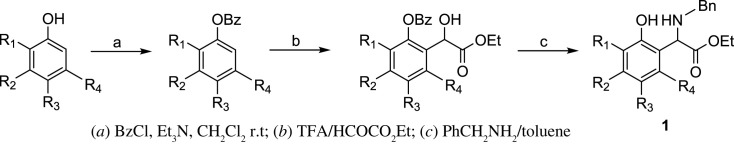
Synthesis of *N*-substituted (ortho-hydroxy)aryl glycine esters.

Key steps in synthetic design involve the construction of a benzofuran-2(3*H*)-one framework and introduction of an aminated chiral centre at the C3 position. These components are considered as important pharmacophores [13,14]. One common approach is the cascade acid-catalysed aza-Friedel-Crafts alkylation and lactonization of phenolic compounds with methyl acetamidoacrylate or its analogues, such as dehydroalanine [18], hippuric acid [19], isatin [20] and azlactone [17]. These compounds have been successfully applied in the synthesis of natural products [21–23]. Another frequently used strategy involves the structural modification of 2-coumaranone derivatives, which can be achieved through direct amination at C3 position under various conditions [24–27]. An alternative option is to establish a double bond or introduce a leaving group on the furan ring, followed by bonding with amino groups through spirocyclization or nucleophilic substitution [28–30]. In addition to phenolic compounds, quinone or imidized phenylglyoxylate can also be used as an aromatic ring moiety to construct the desired skeleton. The fusion of these compounds with furan ring segments begins with amination at the benzylic position through 1,2- or 1,4-nucleophilic addition, followed by intramolecular esterification to generate 3-alkyl-3-*N*-substituted 2-coumaranones [31,32] (scheme 1).

Despite the above-mentioned advances, there is still a need to explore other practical and effective synthetic approaches, particularly focusing on stereoselectivity and stereoisomer separation. In our recent study, we have developed a new method to prepare *o*-quinone methide from (*ortho*-hydroxymethyl)aryl benzoates under mild conditions. This method enabled efficient synthesis of various *N*-substituted (*ortho*-hydroxy)aryl glycine esters **1** from substituted phenols [33]. In this paper, we present a convenient and efficient approach for synthesizing 3-alkyl-3-*N*-substituted aminobenzofuran-2(3*H*)-ones using a cascade Michael addition/lactonization process, taking into account the specific structures of the target molecules.

## 2. Material and methods

The (*ortho*-hydroxymethyl)aryl glycine derivatives or (*ortho*-hydroxyl)phenyl acetic acid ethyl ester (0.25 mmol), acrylonitrile or methyl acrylate (1.5 mmol), Cs_2_CO_3_ (1.0 mmol) and dry toluene (20 ml) were placed in a sealed tube under argon. The sealed tube was stirred at 50°C and thin layer chromatography (TLC) was used to screen the reaction. When TLC showed complete consumption of the reactants, the reaction mixture was cooled to room temperature and diluted with ethyl acetate (100 ml). The solid was filtered, the filtrate was washed with water (2 x 30 ml) and brine (10 ml), and the organic phase was dried over sodium sulfate. After filtration and concentration under reduced pressure, the residue was purified by flash chromatography on silica gel using petroleum ether/ethyl acetate (20 : 1 to 5 : 1 v/v) to afford corresponding products.

UV-vis spectra were recorded using a Unicam SP 800 spectrophotometer. ^1^H and ^13^C nuclear magnetic resonance (NMR) spectra were recorded on a Varian 400 spectrometer. Mass spectra were recorded using a JMX-DX300 spectrometer. Elemental analyses were performed using a Perkin-Elmer 240 microanalyser. Methyl pyropheophorbide-a **1a** was obtained according to the method [31,32]. All chemical reagents were purchased from Merck, Fluka and Aldrich and purified using standard methods.

## 3. Results and discussion

### 3.1. Syntheses of 3-alkyl-3-*N*-substituted aminobenzofuran-2(3*H*)-ones

Structurally, *N*-substituted (*ortho*-hydroxy)aryl glycine esters can be considered as lactone ring opening derivatives of 3-*N*-substituted benzofuran-2(3*H*)-ones. The most commonly used method for synthesizing benzofuran-2(3*H*)-ones involves the lactonization of (*ortho*-hydroxy) aryl acetic acids using either trichlorophosphate or catalytic amount of *p*-toluenesulfonic acid [34,35]. Since *N*-substituted (*ortho*-hydroxy)aryl glycine esters are unstable under strong basic/acidic or high temperature conditions [36], we aimed to investigate new lactonization conditions. Our research focused on identifying suitable inorganic bases and solvents.

The lactonization of (*ortho*-hydroxy)aryl acetic acid ethyl ester (**1a**) was investigated in dry ethanol or toluene using different bases (table 1). The results (table 1, entries 1, 3, 5 and 7) showed that the polar solvent was not suitable for this transformation as the substrate was completely decomposed under basic conditions. When toluene was used as the solvent, different reaction results were observed depending on the type of base. Despite varying reaction conditions, no expected reaction was observed with K_3_PO_4_, K_2_CO_3_ or NaOH as the base. Therefore, further screening of inorganic bases was necessary. To our delight, caesium carbonate was found to be a suitable base, leading to the formation of the desired cyclized product **2a** with a 31% yield. This indicated that Cs_2_CO_3_ should be considered as a suitable base for the generation of 3-*N*-substituted benzofuran-2(3*H*)-one. Based on this result, the reaction conditions were further optimized by adjusting the amount of base, reaction temperature and reaction time (table 1, entries 9–13). The optimal condition was established as using 4.0 equivalents of caesium carbonate and 6 equivalents of methyl acrylate in dry toluene at 50°C for half an hour, which provided a 73% yield of the product **2a**. The chemical structure of the lactone product was confirmed through spectrum analysis, and further evidence was obtained from the formation of α-trisubstituted amine 3 through hydrogenolysis of **2a** and subsequent amidation ring-opening procedures [37].

**Table 1.** Optimization of the reaction conditions for the synthesis of **2a**.

entry	base (equivalent)	solvent	temp	time (h)	yield of **2a** (%)^a^
1	K_3_PO_4_ (5.0)	EtOH	60°C	2	0^b^
2	K_3_PO_4_ (5.0)	toluene	60°C	2	0^c^
3	K_2_CO_3_ (4.0)	EtOH	60°C	2	0^c^
4	K_2_CO_3_ (4.0)	toluene	60°C	2	0^c^
5	NaOH (3.0)	EtOH	40°C	2	0^b^
6	NaOH (3.0)	toluene	40°C	2	0^b^
7	Cs_2_CO_3_ (4.0)	EtOH	60°C	2	0^b^
8	Cs_2_CO_3_ (4.0)	toluene	60°C	2	31
9	Cs_2_CO_3_ (4.0)	toluene	60°C	1	45
10	Cs_2_CO_3_ (2.0)	toluene	60°C	1	20^c^
11	Cs_2_CO_3_ (4.0)	toluene	50°C	1	42
12	Cs_2_CO_3_ (4.0)	toluene	35°C	3	25
13	Cs_2_CO_3_ (4.0)	toluene	50°C	0.5	73^c^

^a^Isolated yield.

^b^Compound **1** was completely decomposed.

^c^Unreacted compound **1** were recovered.

**Scheme 2.**
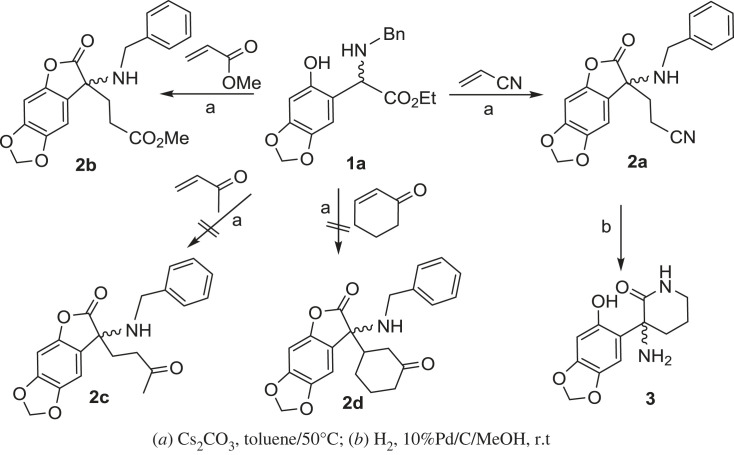
Reactions of *N*-substituted arylglycine esters **1a** with the active methylene compounds.

Compound **1a** was decomposed under all tested conditions in the absence of acrylonitrile. These findings suggested that Michael addition between the α-position of aryl glycine esters and acrylic compounds might be a key step for lactonization. To validate this hypothesis, other α, *β*-unsaturated carbonyl compounds, including butenone (MVK), cyclohex-2-enone, acrylamide and methyl acrylate, were examined using the optimized conditions. When cyclohex-2-enone, butenone and acrylamide were used as Michael acceptors, no lactone formation was observed, except some decomposition of the starting material **1a**. To our delight, when methyl acrylate was used, the lactonization of **1a** afforded **2b** with a better yield of 83% (scheme 2).

**Scheme 3.**
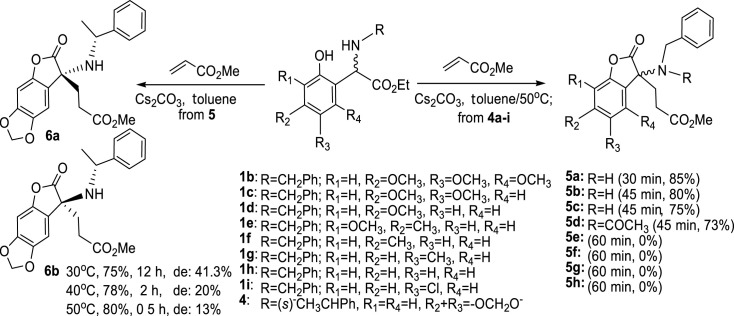
Synthesis of 3-alkyl-3-*N*-substituted aminobenzofuran-2(3*H*)-ones.

With the optimized condition in hand, we subsequently investigated the substrate scope of different *N*-benzyl substituted (*ortho*-hydroxy)phenyl acetic acid ethyl esters by reacting them with methyl acrylate. As shown in scheme 3, the reactivity of the arylglycine derivatives is greatly influenced by the substituent groups on the aromatic ring. When phenyl acetic acid ethyl esters **1b–e** were used, the lactonization reactions proceeded smoothly, leading to products **5a–e** with moderate to good yields (73–85%). All these substrates contained a strong electron-donating group on the aromatic ring, such as an alkoxyl group. However, when substrates **1f–i** were used, in which the alkoxyl group is absent, desired products **5e–h** were not obtained, and a large amount of reactants were recovered. Although substrates **1f** and **1g** have a methyl group at the para- or meta-position of the arylglycine segment, their electron-donating capability is insufficient to initiate the Michael addition and lactonization reactions. This suggests that the presence of a strong electron-donating group in the aromatic ring is crucial for the cascade process.

In this study, we were wondering if we could employ chiral *N*-substituted (*ortho*-hydroxy)phenyl acetic acid ethyl esters to prepare title compounds in a enantioselective manner. We hypothesized that using substrates with significant stereo hinderance could induce stereoselectivity to the cascade process. To test this, we treated *N*-(*R*)-chiral group-substituted (*ortho*-hydroxy)phenyl acetic acid ethyl ester **4**, which consisted of a pair of epimers with a ratio of 1.5 : 1, with Cs_2_CO_3_ and methyl acrylate at various temperatures in dry toluene. Our results showed that the reaction temperature and time significantly influenced the stereoselectivity. Notably, when the reaction was conducted overnight at 30°C, the diastereomeric excess of **6** reached 41.3% which was determined by NMR. Furthermore, we successfully isolated these two diastereoisomers using silica gel chromatography, demonstrating a simple method for preparing chiral 3-*N*-substituted benzofuran-2(3*H*)-ones (scheme 3).

Next, the influence of the number and position of strongly electron-donating substituents on the aromatic ring was investigated. Highest yield and shortest reaction time were obtained from trialkoxyl-substituted phenyl acetic acid ethyl ester **1b**. The reaction results of dialkoxyl-substituted substrates (**1a** and **1c**) were better than those of monoalkxyl-substituted substrates (**1d** and **1e**), indicating that higher electron density on the aromatic ring promoted this cascade process. We also observed slight differences in yields for substrates with two alkoxyl groups on the aromatic ring. Based on the reaction results of **1a** and **1c**, it can be concluded that the bridged dioxymethylene possesses a stronger electron-donating effect compared with two methoxy groups connected at the same position. This could be due to the rigidity of the dioxymethylene structure, which ensures conjugation between the oxygen atoms and the aromatic ring. On the other hand, compounds **1d** and **1e**, which have an extra methyl group in the aromatic ring, were converted to the relevant amino benzofuran-2(3*H*)-one with slightly lower yields. This suggests that weak electron-donating groups cannot promote the reaction. Furthermore, the relative position of the alkoxyl group to the glycine segment is an important factor affecting the structural conversion. The presence of a single methyl group on the phenyl is unable to initiate the reaction, as supported by the results of substrates **1f** and **1g** mentioned above.

### 3.2. The forming mechanism of 3-alkyl-3-*N*-substituted aminobenzofuran-2(3*H*)-one

**Scheme 4.**
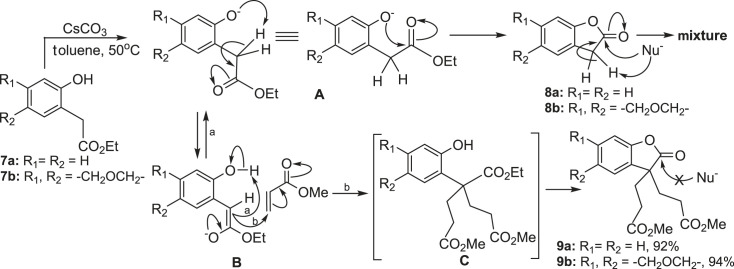
Plausible formation mechanism of 3, 3-disubstituted 2-coumaranone **10a,b**.

To further investigate the reaction mechanism, we first treated phenyl acetic acid ethyl ester **7a,b** under the optimized reaction condition, but without any Michael acceptor. Similar to compound **1a**, these substrates did not form desired 2-coumaranone **8a,b**, but completely decomposed as the reaction time increased. Subsequently, we added three equivalents of methyl acrylate, and 3,3-disubstituted benzofuran-2(3*H*)-one **9a,b** were obtained with high yields. Based on these results, we proposed a possible mechanism for the conversion of **7** to **9**. In this mechanism, phenyl acetic acid ethyl ester **7** first undergoes Michael addition with methyl acrylate to form intermediate **C**, then undergoes intramolecular lactonization to produce 3,3-disubstituted 2-coumaranone **9a,b** (scheme 4).

Compared with the speculated intermediate **C**, which has two bulky substituents at the α-position of the ester group, compound **7** is more likely to undergo lactonization, since the nucleophilic addition reaction between the hydroxyl group and ester group encounters lower steric hindrance. Therefore, we believed that 2-coumaranone **8** might be formed, but it is very unstable and sensitive to alkaline environments, so it completely decomposed after formation. However, 3, 3-disubstituted 2-coumaranone **9** is stable under the same condition, possibly due to the steric hindrance limiting the reactivity of lactone. In practice, phenolate anion **A**, as the preferentially generated conjugated base of **7**, first isomerized into enol anion **B** by proton abstraction from the carbonyl α-position and formed an equilibrium. If there is no Michael reaction acceptor in the reaction system, the phenolate anion will cyclize to 2-coumaranone **8** by intramolecular nucleophilic addition. In the presence of methyl acrylate, Michael adduct **C** is generated through route-b, which is immediately converted to 3, 3-disubstituted 2-coumaranone **9**. 2-coumaranone **8** can easily react with other nucleophiles under basic conditions to produce a complex mixture. For 3, 3-disubstituted 2-coumaranone **9a-b**, the two bulky groups at the 3-position not only occupy the active α-carbonyl, but also form a huge steric hindrance around the carbonyl group at the 2-position, consequently increasing their stability under basic conditions.

### 3.3. The configurations of 3-alkyl-3-*N*-substituted aminobenzofuran-2(3*H*)-ones

The enolate anion derived from phenyl acetic acid ethyl ester **1** could react with ethyl propionate or acrylonitrile in two dominant conformations, **1Ba** and **1Bb**, in which stability mainly came from the hydrogen bond between the hydroxyl and amino groups, as well as the conformational distributions of the bulk substituted groups. Because of the mutual transformation by rotation around the C-N bond of the benzyl group, the two conformations encounter exactly the same spatial circumstance in the Michael addition process; therefore, this reaction generates a pair of equal enantiomers.

**Figure 2.**
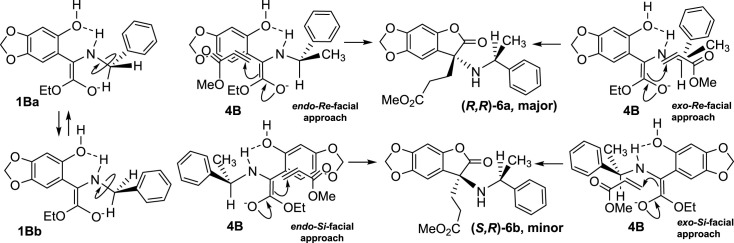
Stereoselective reaction of *N*-chiral group substituted aminoarylglycine ester **4** with methyl acrylate.

Unlike the formation of **1Ba** and **1Bb** mentioned above, the enolate anion formed from compound **4** has only one preferential conformation, **4B**, due to the asymmetry of the C-N bond. The Michael reaction with methyl acrylate can lead to two possible diastereomers, arising from the facial selectivity of either face of the enolate anion plane and *cis-trans* regional selectivity of the enolic double bond. Under this conformation, the Michael receptor can approach from both sides of the molecular plane in the fashion of partial overlap with the aromatic ring (*endo-facial*) or substituted amino group (*exo-facial*). When **4B** is close to the Michael acceptor from the two sides of the aromatic ring plane (*Si*-facial or *Re*-facial), the *p*-orbitals in *π*-systems strongly repel each other, so the *endo-Re*-facial and *endo-Si*-facial approaches are not favourable for Michael addition. Even when nucleophilic addition occurs on the side of the aromatic ring, these reactions do not affect the configurational ratio of the products, because they are far away from the chiral centre. In the process of closing the Michael receptor from the side of the substituted amino group, the steric hindrances in the *exo-Re*-facial and *exo-Si*-facial approaches are different, which is also a major factor that influences the stereoselectivity of the reaction. Compared with that in exo-Si-facial manner, nucleophilic addition in *exo-Re*-facial manner encounters less spatial resistance from the methyl group at the chiral centre, and thus affords the major product with (*R, R*)-configuration. When addition reaction proceeds in *exo-Si*-facial approach, the crowded circumstance between methyl acrylate and phenyl decreases the reaction rate, leading to the formation of (*S, R*)-2-coumaranone in a relatively low yield (figure 2).

According to the analysis above, chirality at the α-position of *N*-substituted (*ortho*-hydroxy)aryl glycine esters completely racemized after the formation of enolate anions in this cascade reaction; therefore, the ratio of the two enantiomers in the substrate does not affect the diastereomeric excess of the final product. The absolute configuration and size of the chiral group attached to nitrogen are important factors in determining the diastereoselectivity of the title compound. It is reasonable to believe that if different chiral *N*-substituted (*ortho*-hydroxy)aryl glycine esters are used, chiral 3-alkyl-3-*N*-substituted aminobenzofuran-2(3*H*)-ones could be obtained as a pair of epimers through regular chromatographic separation.

## 4. Conclusion

In summary, we reported an efficient and practical method for the preparation of 3-alkyl-3-*N*-substituted aminobenzofuran-2(3*H*)-ones from *N*-substituted (*ortho*-hydroxy)aryl glycine esters via a novel Cs_2_CO_3_-mediated cascade reaction. Detailed mechanistic studies for this transformation suggested that in this cascade event, a Michael addition occurs first to produce a tri-substituted acetic acid ethyl ester intermediate, followed by intramolecular lactonization to construct the skeleton of benzofuran-2(3*H*)-one. The substrate scope of this cascade reaction is widespread and includes different aryl glycine esters and *α*, β-unsaturated carbonyl compounds. Stereoselective results, such as the determination of the configuration and separation of epimers, strongly illustrate the potential use of this method in the preparation of enantiopure 3-alkyl-3-*N*-substituted aminobenzofuran-2(3*H*)-ones. This new strategy provides a valuable synthetic route for obtaining new 3-aminated-2-benzofuranones with potential further applications. Enhancing the stereoselectivity of this method and its application to the synthesis of natural products or other interesting chiral compounds is in progress in our laboratory.

## Data Availability

The datasets supporting this article have been uploaded as part of the electronic supplementary material [38].
